# Isoliquiritigenin induces apoptosis of human bladder cancer T24 cells via a cyclin-dependent kinase-independent mechanism

**DOI:** 10.3892/ol.2021.12529

**Published:** 2021-02-09

**Authors:** Lingling Si, Xinhui Yang, Xinyan Yan, Yanming Wang, Qiusheng Zheng

Oncol Lett 14: 241-249, 2017; DOI: 10.3892/ol.2017.6159

Subsequently to the publication of this paper, an interested reader drew to the authors’ attention that, in Fig. 10, lanes 2–3 in the Bcl-2 panel appeared to be strikingly similar to lanes 1–2 in the Caspase-9 panel.

The authors have re-examined their data and realized that Fig. 10 was assembled incorrectly; essentially, the data for the Bcl-2 and Caspase-9 bands, which were similar in actual appearance, were confused during the final compilation of the Figure. The revised version of Fig. 10, containing the correct data for the Caspase-9 experiment, is shown below. The authors regret the error that was inadvertently made in the preparation of the Figure, and confirm that this error did not seriously affect the conclusions reported in the paper. The authors are grateful to the editor of *Oncology Letters* for allowing them the opportunity to publish a Corrigendum, and all the authors agree to this Corrigendum. Furthermore, they apologize to the readership for any inconvenience caused.

## Figures and Tables

**Figure 10.**
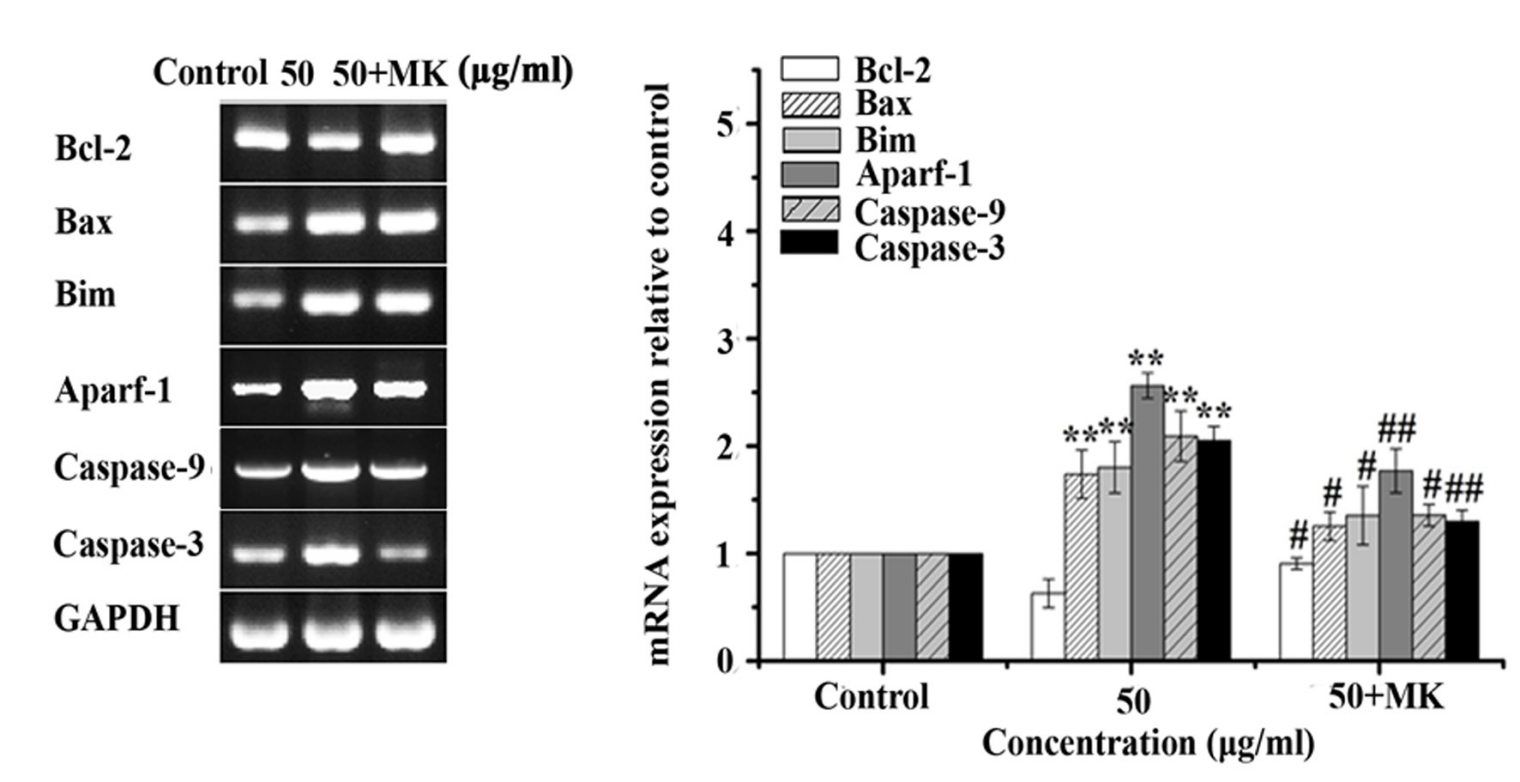
MK-8776 antagonizes the increases in Bax, Bim, Apaf-1, caspase-9 and caspase-3 mRNA expression, and the decrease in Bcl-2 mRNA expression in T24 cells. T24 cells pretreated or not with MK-8776 were treated with ISL at 50 µg/ml and mRNA expression was determined using the reverse transcription-polymerase chain reaction and agarose gel electrophoresis. Quantification results are presented as the mean ± standard deviation of three independent experiments. **P<0.01 vs. untreated control group cells; ^#^P<0.05, ^##^P<0.01 vs. 50 µg/ml ISL-treated group cells. Bax, Bcl-2-associated X protein; Bim, Bcl-2-interacting mediator of cell death; Apaf-1, apoptotic protease-activating factor-1; Bcl-2, B-cell lymphoma 2; ISL, isoliquiritigenin.

